# ArachnoServer: a database of protein toxins from spiders

**DOI:** 10.1186/1471-2164-10-375

**Published:** 2009-08-13

**Authors:** David LA Wood, Tomas Miljenović, Shuzhi Cai, Robert J Raven, Quentin Kaas, Pierre Escoubas, Volker Herzig, David Wilson, Glenn F King

**Affiliations:** 1 Queensland Facility for Advanced Bioinformatics, The University of Queensland, St Lucia, QLD 4072, Australia; 2 ARC Centre of Excellence in Bioinformatics, The University of Queensland, St Lucia, QLD 4072, Australia; 3 Institute for Molecular Bioscience, The University of Queensland, St Lucia, QLD 4072, Australia; 4 Queensland Museum, Brisbane QLD 4101, Australia; 5 Institut de Pharmacologie Moléculaire et Cellulaire, CNRS, 06560 Valbonne, France

## Abstract

**Background:**

Venomous animals incapacitate their prey using complex venoms that can contain hundreds of unique protein toxins. The realisation that many of these toxins may have pharmaceutical and insecticidal potential due to their remarkable potency and selectivity against target receptors has led to an explosion in the number of new toxins being discovered and characterised. From an evolutionary perspective, spiders are the most successful venomous animals and they maintain by far the largest pool of toxic peptides. However, at present, there are no databases dedicated to spider toxins and hence it is difficult to realise their full potential as drugs, insecticides, and pharmacological probes.

**Description:**

We have developed ArachnoServer, a manually curated database that provides detailed information about proteinaceous toxins from spiders. Key features of ArachnoServer include a new molecular target ontology designed especially for venom toxins, the most up-to-date taxonomic information available, and a powerful advanced search interface. Toxin information can be browsed through dynamic trees, and each toxin has a dedicated page summarising all available information about its sequence, structure, and biological activity. ArachnoServer currently manages 567 protein sequences, 334 nucleic acid sequences, and 51 protein structures.

**Conclusion:**

ArachnoServer provides a single source of high-quality information about proteinaceous spider toxins that will be an invaluable resource for pharmacologists, neuroscientists, toxinologists, medicinal chemists, ion channel scientists, clinicians, and structural biologists. ArachnoServer is available online at http://www.arachnoserver.org.

## Background

The realisation that many venomous creatures possess a complex repertoire of protein toxins with pharmacological, pharmaceutical, and agrochemical applications has led to an exponential increase in the number of new toxins being discovered [1]. Several electronic databases have been established to capture information about subsets of these toxins, such as ConoServer [2] and SCORPION2 [3], which provide information about toxins from marine cone snails and scorpions, respectively. Other databases provide information about all groups of animal toxins, such as Tox-Prot [4] and the Animal Toxin Database (ATDB) [5]. These databases allow cross-comparison of toxins from different animal groups but the individual toxin records typically lack the rich information content of manually curated databases.

Spiders are by far the largest group of venomous animals, with more than 40,000 extant species [6], and they maintain the largest repertoire of pharmacologically active peptides [7]. Some spider toxins have been used for almost two decades as pharmacological tools to probe the structure and function of ion channels and cell-surface receptors [8,9]. Other spider toxins are being developed as bioinsecticides [10-12] or providing leads for the development of drugs to treat a diverse range of pathophysiological states such as inflammatory and neuropathic pain [13], cardiac arrhythmia [14], and erectile dysfunction [15]. Remarkably, despite the incredible chemical diversity of spider venoms, there are no curated databases that deal specifically with proteinaceous toxins from these animals. Here we introduce ArachnoServer, a database designed to provide detailed and easily accessible information on the sequence, three-dimensional structure, and biological activity of spider toxins.

## Construction and content

### Data sources and integration

A non-redundant set of publicly available spider toxin sequences was sourced from Swiss-Prot [16] and GenBank [17], while experimentally determined protein structures were obtained from the Protein Data Bank [18]. Taxonomic information on spiders (Araneae) was retrieved from the World Spider Catalog [6], which has the advantage of providing more up-to-date taxonomy than NCBI, as well as historic taxonomic information that can be used in database searches. As the World Spider Catalog is under constant revision, care was taken to ensure future compatibility of the ArachnoServer data model with this catalog. Each species in ArachnoServer is assigned a unique life science identifier (LSID) which is linked to the equivalent LSID in an electronic version of the World Spider Catalog. In this way, the ArachnoServer database is automatically linked to taxonomic updates within the World Spider Catalog. High-resolution images of spiders, which are provided without restriction for academic use, were obtained from private collectors and have been used only in cases where both the species and sex could be verified.

A key feature of ArachnoServer is the molecular target ontology. The majority of spider toxins act on ion channels and cell-surface receptors [19], and consequently we developed a new molecular target ontology based on the channel and receptor subtype definitions and nomenclature recommended by the International Union of Basic & Clinical Pharmacology [20]. Additional descriptors were added for toxins that target invertebrate ion channels and receptors, cytolytic toxins, lectins, toxins that target enzymes, and toxins with enzymatic activity. This new ontology allows for the first time the retrieval of spider toxin information based on a precise definition of toxin specificity. For example, this ontology makes it facile to search the database for toxins that target a specific subtype of vertebrate ion channel or receptor. Finally, spider toxins often present diverse posttranslational modifications, and these are described in ArachnoServer using an ontology derived from ConoServer [2].

### Data curation

Where appropriate, extra information from the literature was added to each imported toxin record and additional citations were incorporated using the PubMed EFetch service. Year of discovery was carefully curated according to the date on which a manuscript describing the sequence was first submitted for publication, or the date when the toxin sequence was first submitted to GenBank or SWISS-PROT or appeared in the patent literature; this information is often incorrect in the source databases. Toxins were assigned recommended names according to the recently described rational nomenclature for spider toxins [1] and all available literature synonyms were also attached to each toxin record.

ArachnoServer provides the ability to curate information specific to a particular toxin as well as data that are generically applicable. For example, curators can create, edit, or delete symbols and names for posttranslational modifications. Data sets available for curation include lists of posttranslational modifications, biological activities, sequence features (e.g., signal and propeptide sequences), the molecular target ontology, common names and images of spiders, and even the units used to describe LD_50_, PD_50_, and IC_50 _values. The types of experimental evidence for disulfide bonds and toxin pharmacophores (e.g., experimentally determined, by homology, or predicted) can also be managed by the curation team. This inherent flexibility should allow the database to grow in both size and functionality as the knowledge base about spider toxins continues to expand.

### Application architecture

ArachnoServer is a Java Spring Model View Controller (MVC) application that uses a Hibernate Object Relational Mapping (ORM) layer to a MySQL database. All database tables are mapped to Plain Old Java Object (POJO) serializable beans. Hibernate manages the writing, updating, and deleting of data from the database tables according to changes in the POJO data, instigated by events triggered from the view and propagated through to the Spring service layer. Using the Spring architecture with ORM mapping, the application and data model can be easily extended or modified, as changes to the data model do not require SQL changes (Hibernate transparently creates all SQL statements using mapped POJOs).

## Utility and discussion

### Interface and visualisation

**Figure 1:**
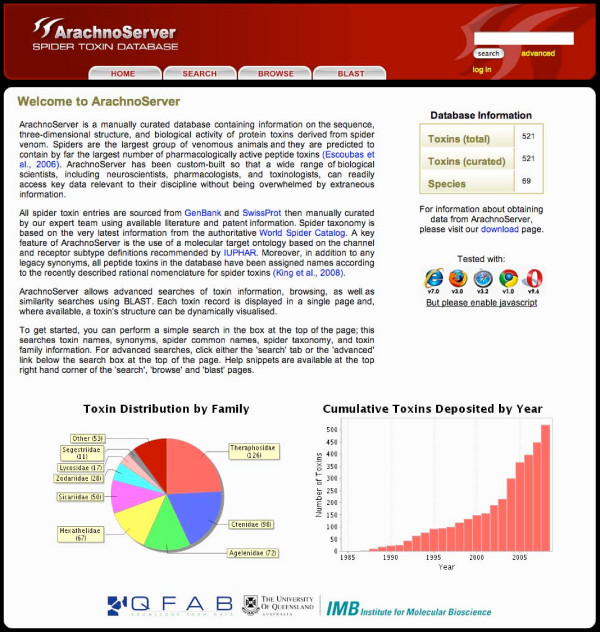
**ArachnoServer interface**. The front page of ArachnoServer provides a brief introduction to the database, information about performing searches, and details of the total number of available toxins, their taxonomic distribution, and year of discovery. Note that basic searches can be performed from every page within the database using the search bar in the ArachnoServer banner.

ArachnoServer is presented using a graphically designed CSS skin that can be easily swapped for other skins in future releases if required. The front page provides statistics about the total number of toxins available, their taxonomic distribution, and year of discovery (Fig. 1). Individual toxin records can be accessed through basic or advanced searches or via the browse function, all of which are described below.

**Figure 2:**
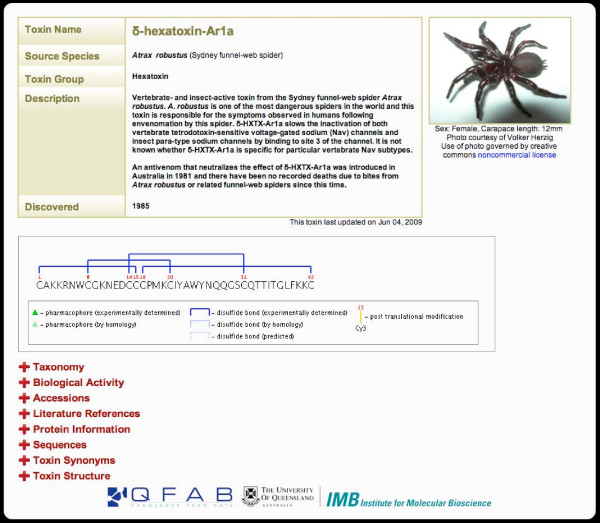
**Example of a Spider Toxin Record (STR)**. STR for δ-hexatoxin-Ar1a, the lethal toxin from the Sydney funnel-web spider *Atrax robustus*. The top section of the STR provides a summary of the toxin's activity, the source species, and year of discovery. The dynamically generated toxin image displays the mature toxin sequence and, where available, its disulfide-bond framework, pharmacophore residues, and posttranslational modifications. Below this image are additional user-expandable sections that provide information about the toxin's molecular target, phyletic specificity, and 3D structure, as well as literature references, toxin synonyms, and both current and historic taxonomy of the source species. Clicking the spider photo yields a downloadable high-resolution image.

The spider toxin record (STR) card summarises all information about a toxin in a clean and intuitive interface (Fig. 2). A description of the toxin's biological activity is provided at the top of the page followed by a dynamically generated image that displays the mature toxin sequence along with information about disulfide bond connectivities, pharmacophore residues, and post-translational modifications. Mousing over the image displays additional information, including the position in the sequence and full residue name. The expandable sections below the image were carefully designed to allow biologists working in different disciplines to find the desired information without being distracted by extraneous data. For example, pharmacologists interested in the molecular target of the toxin but not other details can click on the *Biological Activity *tab in order to find this specific information, whereas structural biologists and medicinal chemists may be more interested in accessing details of the structure via the *Toxin Structure *section, which allows the structure to be viewed in interactive 3D mode via a Jmol applet http://www.jmol.org. For all toxins, a colour-coded image is displayed in the *Protein Information *section that provides a quick overview of the signal sequence, propeptide, and mature toxin regions. Other tabs provide information on the spider taxonomy (*Taxonomy*), links to external databases (*Accessions*), links to relevant literature (*Literature References*), sequences in FASTA format (*Sequences*), and alternative toxin names (*Toxin Synonyms*).

### Basic and advanced searches

ArachnoServer supports basic searches and advanced searches. Basic searches query toxin names (including synonyms), taxonomic information (including historic taxonomy), common names of spiders, and toxin families. Basic searches can be performed from every page using the search bar in the ArachnoServer banner.

**Figure 3:**
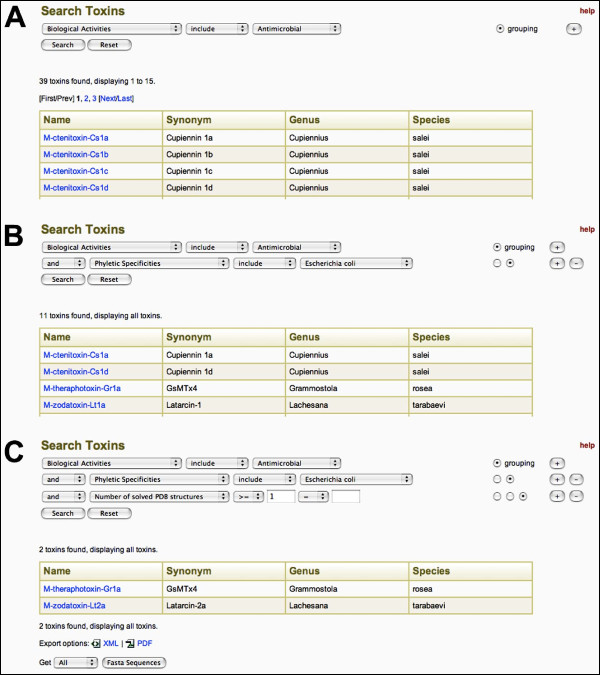
**Advanced searches in ArachnoServer**. An example of the advanced search feature in ArachnoServer, which can be used to search the database using a *combination *of criteria (see text for details). Only the first four toxins from the search results are displayed in (A) and (B) for the sake of brevity. Clicking on the toxin name (highlighted in blue) opens up the corresponding spider toxin record. Results can be exported in XML, PDF, or FASTA format. A help button is available in the top right corner of the search page.

A key feature that distinguishes ArachnoServer from other toxin databases is the advanced search feature (Fig. 3). Context-specific fields are dynamically arranged on the page, and search keys populated in drop-down lists of relevant data from the database. Search clauses can be grouped, then joined using boolean operators. Most database fields can be searched, including all basic search fields, as well as additional features such as biological activity, posttranslational modifications, literature references, and counts of various data fields (e.g., the number of disulfide bonds in a toxin). Results from both basic and advanced searches are displayed as paginated tables that can be exported in XML or PDF format. FASTA sequences for toxins from any set of search results can also be exported in plain text format.

The advanced search feature provides a convenient way for scientists to locate records of immediate interest. For example, a medicinal chemist or microbiologist focused on infectious diseases might be particularly interested in toxins that have antimicrobial activity. Using the advanced search feature to search for toxins with antimicrobial activity reveals that the database currently contains 39 such toxins (Fig. 3A). However, this search could be restricted according to many different criteria, such as the year of discovery, activity against specific bacteria or parasites, or the number of disulfide bonds and posttranslational modifications in the toxin. For example, adding a requirement for activity against *Escherichia coli *limits the search result to 11 toxins (Fig. 3B). The medicinal chemist might be particularly interested in the subset of these toxins for which a 3D structure has been determined. Adding this criterion limits the search result to two toxins (Fig. 3C), for which the 3D structure can then be viewed from the individual spider toxin records.

### Browsing

**Figure 4:**
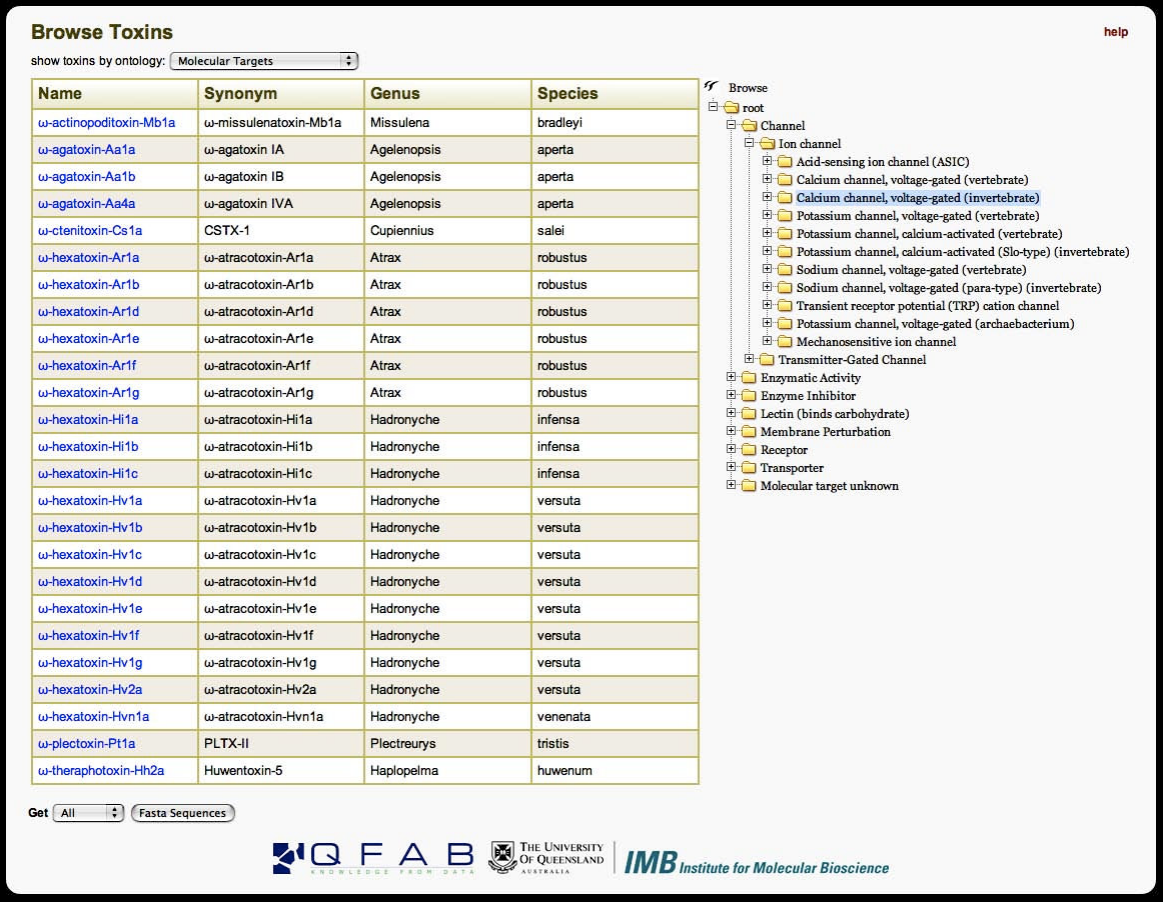
**Browsing based on molecular ontology**. An example of the browse molecular ontology function in ArachnoServer, which in this example has been used to locate all toxins in the database that are known to act on invertebrate voltage-gated calcium channels.

Browsing of the toxin records is also possible through the browse page. Dynamic trees are populated with folders of toxins that match the grouping criteria. Users can browse the spider taxonomic tree and view all toxins for a given taxon. Similarly, the molecular target ontology and posttranslational modifications can be browsed. Fig. 4 shows an example where the molecular ontology browse function has been used to locate all toxins in the database that are known to target invertebrate voltage-gated calcium channels. Note that a complete list of FASTA sequences can be obtained from browse results.

### Sequence similarity searches

Similarity searches using BLAST [21] are available through the BLAST page and also from each of the sequence records in the STR page. Both protein and nucleic databases can be searched using the programs blastn, blastp, blastx, tblastn, and tblastx. Hits from toxins in the database are linked directly from the BLAST result page to the STR cards in ArachnoServer, and, where available, back to the original sources in GenBank or Swiss-Prot.

## Conclusion

ArachnoServer is a web-based resource that provides comprehensive information about protein toxins from spider venoms. The combination of specific classification schemes and a rich user interface allows researchers to easily locate and view information on the sequence, structure, and biological activity of these toxins. This manually curated database will be a valuable resource for both basic researchers as well as those interested in potential pharmaceutical and agricultural applications of spider toxins.

## Availability and requirements

ArachnoServer is freely available at http://www.arachnoserver.org. ArachnoServer is platform independent and supports most browsers including Firefox (Version 3 and higher), Safari (Ver. 3.2 and higher), Google Chrome (Ver. 1 and higher), Internet Explorer (Ver. 7 and higher), and Opera (Ver. 9.6 and higher). A java plug-in is required in order to view 3D protein structures.

## Authors' contributions

GFK and DLAW devised the ArachnoServer concept and wrote the manuscript. All authors were involved in formulating specifications, application design, and testing. DLAW was responsible for data integration, database design, and application implementation with assistance from TM and SC. SC also worked on application architecture. GFK and QK developed the molecular target and posttranslational modification ontologies, respectively. RR was responsible for integration of taxonomy from the World Spider Catalog. DW, VH, and GFK were responsible for database curation. PE was primary beta tester. All authors read the final manuscript
